# Outcomes of Robotic Surgery in a Single-institution, High-volume Hepatobiliary Oncology Unit

**DOI:** 10.1007/s13193-024-01873-6

**Published:** 2024-01-16

**Authors:** Kunal Nandy, Shraddha Patkar, Gurudutt Varty, Tanvi Shah, Mahesh Goel

**Affiliations:** Division of Hepatobiliary Surgical Oncology, Department of Surgical Oncology, Tata Memorial Hospital, Homi Bhabha National Institute, Parel, Mumbai, Maharashtra India

**Keywords:** Robotic surgery, Hepatectomy, Cholecystectomy, Radical, Metastatectomy

## Abstract

Hepatobiliary surgery has traditionally been performed via an open approach. With the advent of robotic surgery, the minimal access approach in hepatobiliary oncology has gained impetus due to its technical superiority and favorable learning curve over laparoscopy. We present our experience with the Da Vinci Xi system in hepatobiliary oncology. This is a retrospective study from a prospectively maintained database. All patients who underwent surgery between June 2015 and July 2023 for suspected gallbladder cancer and primary or metastatic liver tumors were included. After excluding all inoperables and conversions, a total of 92 patients were included for analysis. There was a conversion rate of 15.6% (17 of 109 patients). Sixty-four (69.6%) patients underwent surgery for gallbladder-related pathologies that included 39 (60.9%) radical cholecystectomies, 24 (37.5%) simple cholecystectomies, and 1 (0.01%) revision cholecystectomy. Twenty-eight patients underwent surgeries for primary or metastatic liver tumors, which included 25 (92.9%) minor and 2 (7.1%) major hepatectomies. Significant morbidity (Clavien–Dindo grade III or more) was seen in 8 (8.6%). There was no postoperative mortality. In the group with gallbladder cancer, the median lymph nodal yield was 7 (2–22) in patients who underwent lymph nodal dissection. The median follow-up was 63.9 (0.49–100.67) (IQR = 37.76) months. The 5-year OS and DFS were 76.4 and 71.3%, respectively. Robotic hepatobiliary surgery is feasible and can be performed safely after adequate training. Patient selection is of utmost importance and is the key to establishing a robust robotic hepatobiliary oncosurgery program.

## Introduction

Hepatobiliary surgery has traditionally been regarded as one of the most complex surgeries due to its associated morbidity and mortality. Traditionally, open surgery has been regarded as the standard approach. In recent years, the use of laparoscopy has increased because of its advantages of lesser postoperative pain and a shorter hospital stay without compromising oncological outcomes as compared to open surgery [1, 2]. Laparoscopic hepatobiliary surgery has a steep learning curve, and procedures are technically demanding. However, with the advent of advanced liver parenchymal transection equipment and improved optical systems, there has been an increase in pursuing a minimal access approach in hepatobiliary surgery [3]. With the robotic system, the limitations of laparoscopy, like the instrument’s range of motion and the surgeon’s hand movements, are well controlled while performing complex hepatobiliary surgery. Robotic surgery has evolved immensely in the past two decades, and with the Xi modification of the Da Vinci system introduced in 2014, there have been many technical advantages to the operating surgeon, thus resulting in ease of operation, which then translates into better outcomes for the patient. We hereby report our experience of patients who underwent robotic hepatobiliary surgeries using the Da Vinci Xi system.

## Materials and Methods

This is a retrospective study of a prospectively maintained database of all patients who underwent robotic surgery between June 2015 and July 2023 for suspected gallbladder cancer and primary or metastatic liver tumors. All surgeries were performed on the robotic Da Vinci Xi system. The primary surgeons (MG and SP) performing robotic surgeries underwent a Da Vinci-certified training program in the USA and South Korea, respectively, before starting robotic surgery.

The decision regarding offering robotic surgery to the patients was taken by a multidisciplinary tumor board involving trained, experienced hepatobiliary surgeons, medical and radiation oncologists, and radiologists. Only patients with early disease based on radiology were offered a robotic approach. Only small intraluminal lesions in the gallbladder with no or minimal hepatic infiltration and no radiological periportal lymphadenopathy were offered robotic surgery.

Preoperative data that were recorded included age, sex, body mass index (BMI), American Society of Anesthesiologists (ASA) classification, comorbidities, previous abdominal surgery, and the presence of chronic liver disease. Intraoperative parameters such as operative time, blood transfusion, use of inflow occlusion, duration, and conversion rate were noted. Operative time was defined as the time from the time of port insertion to closure. Docking time was defined as the time from the first port insertion to the docking of the robot. Procedures started on a robotic platform and later converted to an open approach were considered conversions. Postoperative complications, length of stay, readmission within 30 days, and mortality were documented. Postoperative complications were recorded using the Clavien–Dindo classification up to 90 days after surgery [4]. Grade III or more complications were considered significant morbidity.

The pathological report was noted, and resection margins were defined as R0 resection when tumor distance from the margin was greater than 1 mm, R1 resection when tumor distance from the margin was less than 1 mm, and R2 resection upon the presence of macroscopic tumor at the margin.

### Surgical Techniques

The patient was positioned in reverse Trendelenburg up to 45°, and a slight lateral tilt was given depending on the side of the liver under focus.

**Fig. 1:**
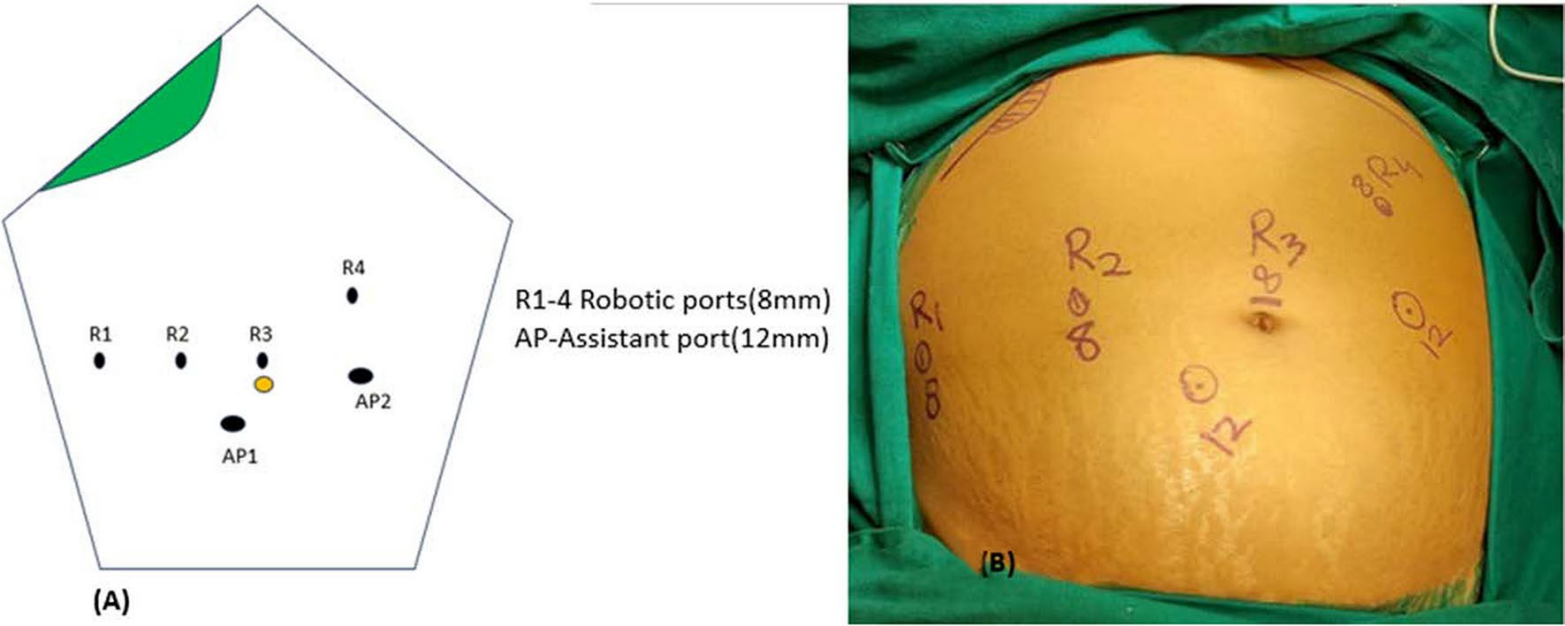
**A, B** Robotic port position (R1 to R4, 8 mm) with assistant ports (AP, 12 mm)

Three ports were placed along a straight line from above the right anterior superior iliac spine to the umbilicus. The procedure was started with a supraumbilical pneumoperitoneum created by open technique and an 8-mm robotic port (R3) inserted. The other ports were placed under vision using a 30° camera after creating pneumoperitoneum. The fourth port (R4) was placed between the R3 port and the left costal margin above the straight line joining the first three ports. One 12-mm assistant port is inserted between R2 and R3, and another between R3 and R4 if required. The camera is inserted in R2 with working arms on either side (Fig. 1). A combination of monopolar scissors, bipolar, prograsp, and needle holders were commonly used during the surgeries.

**Fig. 2:**
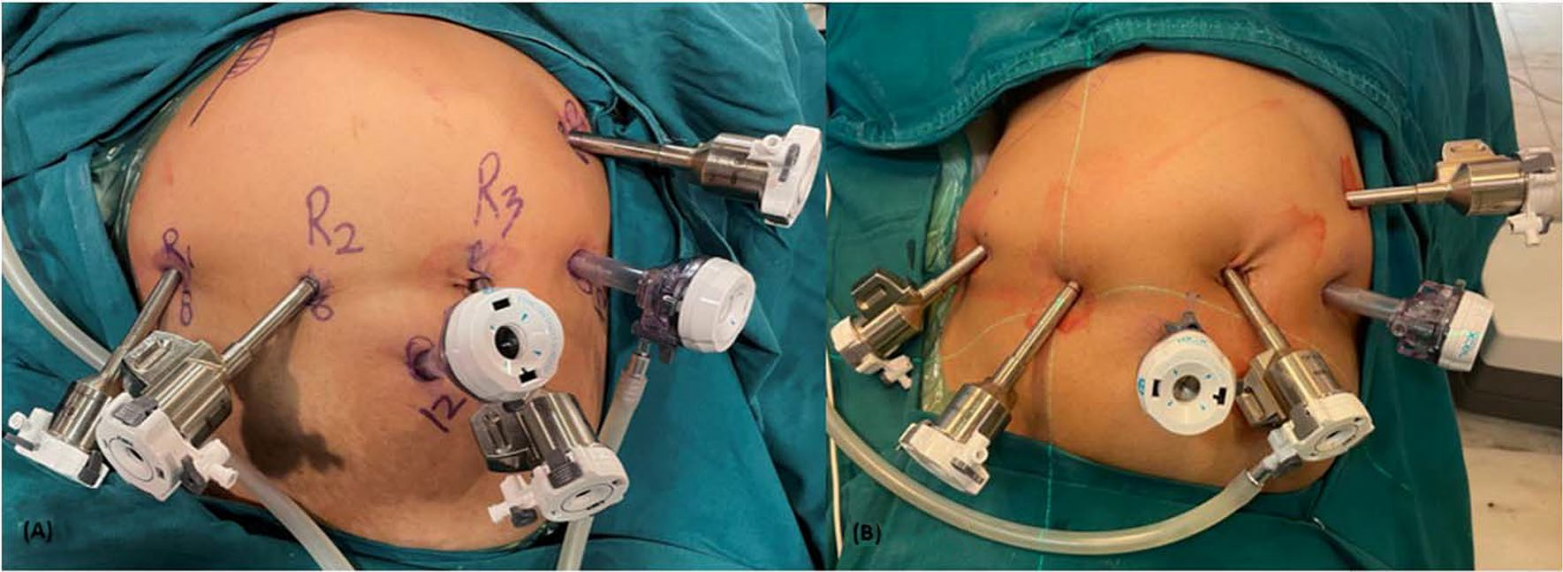
**A** Port positions of robotic (8 mm) and assistant ports (12 mm). **B** Laser pointer in the overhead boom was used to center the boom over the camera port (R2)

**Fig. 3:**
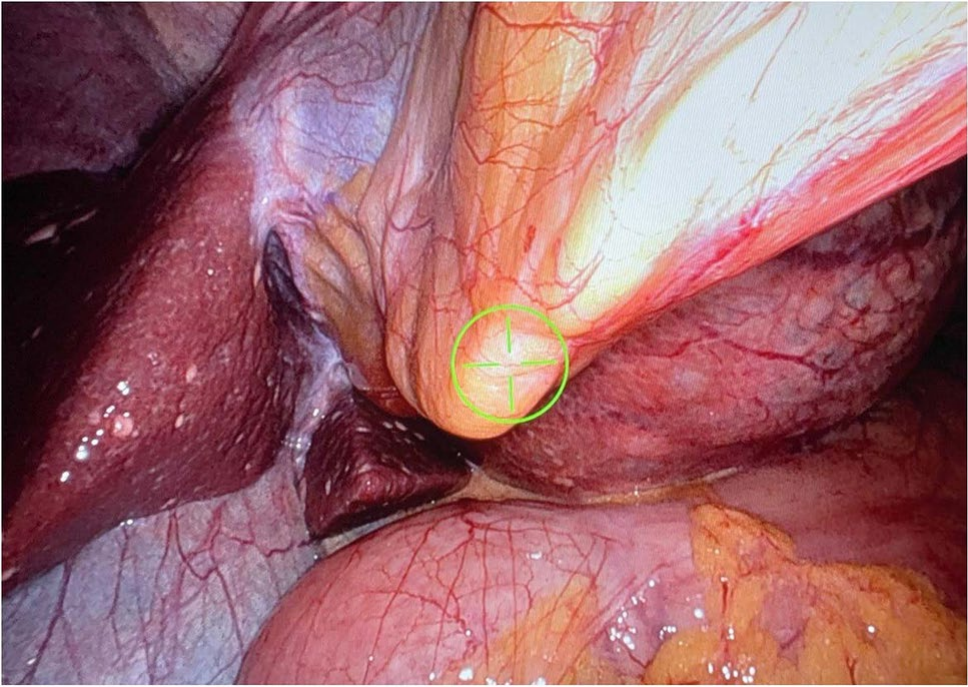
Targeting of operative anatomy over the liver (in this case planned for a left lateral sectionectomy)

Before docking, the patient’s anatomy was selected on the patient cart as upper abdomen, left side, which is defined as per the position of the robot relative to the patient. Robotic arms were then deployed. A laser pointer in the overhead boom was used to center the boom over the camera port (R2) (Fig. 2). Robotic arms were docked. The camera was inserted in R2, and targeting was done, followed by the insertion of the rest of the instruments. Targeting helps in the automatic alignment of the other arms and the overhead boom as per the preset configuration (Fig. 3).

For radical cholecystectomy, the falciform ligament was hitched to the anterior abdominal wall by a suture, and the duodenum was retracted caudally to open the porta hepatis. A calots dissection is performed to identify and clip the cystic duct and artery. A complete hepatoduodenal lymph nodal dissection (stations 8, 12, and 13) is completed, safeguarding the common bile duct, hepatic artery, portal vein, and branches. The gallbladder, along with a 2.5–3-cm liver wedge, is resected using a combination of monopolar and bipolar energy. A similar procedure was done for revision and a simple cholecystectomy. After completion of a simple cholecystectomy, gallbladder was sent for a frozen section. Periportal lymph node dissection and liver wedge resection were done if proven to be malignant.

**Fig. 4:**
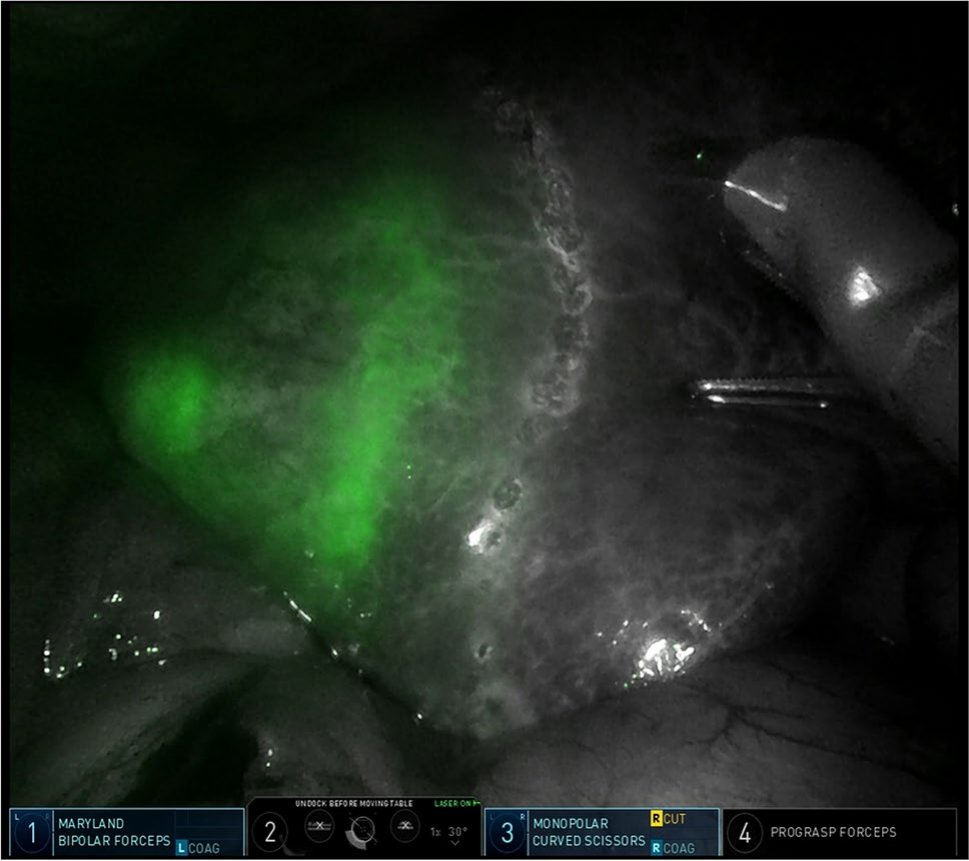
Tumor identification using ICG and resection marking, taking an adequate margin

For liver resection, portal dissection was done first to achieve inflow control. For major hepatectomies, the inflow branches of the portal vein and hepatic artery were divided, and parenchymal transection was done using a combination of monopolar and bipolar energy. For lateral sectionectomies, all pedicles to the left of the falciform ligament, or ligamentum teres, were carefully delineated and transected with clips or a vascular stapler. For non-anatomical resections, the porta hepatis was looped, and parenchymal transection was done, taking adequate margins. For parenchymal transection, a combination of robotic bipolar energy forceps, monopolar forceps, and bedside assistants using harmonic scalpels was used. For vessels < 7 mm, a combination of these energy devices was useful, and for intrahepatic vessels > 7 mm, clips were used. The Pringle maneuver was done in selected patients using a 12-Fr Foley catheter. For intraoperative tumor identification, preoperative intravenous indocyanine green was given at a dose of 0.2 mg/kg at least 5 days before the day of surgery (Fig. 4).

### Statistics

Demographic data and clinical outcomes were analyzed. The descriptive analysis included mean and standard deviation in continuous variables, while categorical and ordinal variables were reported as counts with proportions. Disease-free survival (DFS) was calculated from the day of surgery until the date of recurrence or death, and overall survival (OS) was calculated from the date of surgery until death or the last follow-up. Survival is depicted by Kaplan–Meir curves. Statistical analyses were performed using SPSS Statistics version 24.0 (IBM, Armonk, NY, USA).

## Results

A total of 838 and 1447 gallbladder surgeries were performed in the unit during the period. Of the 838 hepatectomies performed, 780 were open, 22 were done by laparoscopic approach, and 36 were offered robotic surgery. Similarly, out of 1447 patients who underwent surgeries for suspected gallbladder pathologies, 75 were offered a robotic approach, and the rest underwent open surgery.

A total of 113 patients were offered robotic surgery. Out of 113 patients, 4 were declared inoperable given distant metastasis detected intraoperatively. There was a conversion rate of 15.6% (17 of 109 patients) (Table 1).

**Table 1:** Elaborating reasons for conversion

Gallbladder surgery	9
Biliary reconstruction	2
Bleeding	1
Iatrogenic injury	3
Adhesions	2
Inadequate retraction	1
Liver surgery	**8**
Bleeding	2
Instrument failure	2
Need for intestinal resection and anastomosis	2
Lesion could not be localized	1
Inadequate retraction	1

Hence, 92 patients were included in the analysis. The demographic and general characteristics of the patients are illustrated in Table 2.

**Table 2:** Demographic details, surgical details, and outcomes

Parameters	Results
Age	55 (17–76) years
Sex (male:female)	41:51
BMI	24.2 (13.3–38.6) kg/m^2^
ASA (I: II:III)	47:39:6
**Gallbladder pathology**	**64 (69.6%)**
Radical cholecystectomy	39 (60.9%)
Simple cholecystectomy	24 (37.5%)
Revision cholecystectomy	1 (0.01%)
Median blood loss	150 (10–1200) ml (IQR = 275 ml)
Median duration	240 (40–525) min (IQR = 120 min)
Median hospital stay	3 (1–18) days (IQR = 2 days)
Median ICU stay (in days)	1 day
Clavien-Dindo complication (> III)	3
Mortality	0
Malignant pathology	40 (62.5%)
Benign pathology	24 (37.5%)
**Liver pathology**	**28 (30.4%)**
** Major hepatectomy**	**2 (7.1%)**
Left hepatectomy	1
Right hepatectomy	1
** Minor hepatectomy**	**25 (92.9%)**
Left lateral hepatectomy	20
Non-anatomical resection	5
Median blood loss	300 (50–1200) ml (IQR = 432.50 ml)
Median duration	270 (130–690) min (IQR = 200 min)
Median hospital stays	6 (2–25) days (IQR = 5.5 days)
Clavian–Dindo complication (> III)	5
Median ICU stay (in days)	1 (1–2)
Mortality	0
**Primary liver tumor**	
HCC	10 (35.7%)
IHCC	1 (3.6%)
Benign (IgG-related pathology)	1 (3.6%)
**Metastatic liver tumors**	
Colorectal primary	14 (50%)
NET	1 (3.6%)
Breast	1 (3.6%)

*ASA*, American Society of Anesthesiologist; *HCC*, hepatocellular carcinoma; *IHCC*, intrahepatic cholangiocarcinoma; *NET*, neuroendocrine tumor; *IQR* interquartile range; *ICU*, intensive care unit

Among the 64 patients operated on for gallbladder pathology, a significant complication (> Clavein–Dindo grade III) was seen in 3 (4.6%); 2 patients had gallbladder fossa collection that was drained, and one patient was re-explored on postoperative day 2 for jejunal perforation.

Out of 28 patients operated on for liver pathology, 5 (17.8%) had significant complications (> Clavein–Dindo Grade III). One patient who had undergone right hepatectomy had a bile leak detected on POD8, which was managed with PTBD insertion; one patient who had undergone synchronous anterior resection had an anastomotic leak, which was managed by re-exploration for diversion ileostomy; 2 patients had postoperative bed collection, which was drained; and one patient had prolonged ileus, which was managed with gastric decompression. There was no postoperative mortality.

There was no statistically significant difference in outcomes between minor and major hepatectomies (Table 3).

**Table 3:** Major vs. minor hepatectomy

Parameters	Major hepatectomy	Minor hepatectomy	*p* value
Total	4	32	
Conversion	2	6	0.156
Blood loss (ml)	500 (400–600)	300 (50–1200); IQR = 395	0.206
Duration (min)	545 (400–690)	260 (45–630); IQR = 180	0.481
Hospital stays (days)	14 (9–19)	5.5 (2–25); IQR = 5	0.175
ICU stay (days)	1 (1–2)	1 (1–2)	0.382
Major complication (Clavien–Dindo ≥ 3)	1	4	0.307

*ICU*, intensive care unit

### Oncological Outcomes of Gallbladder Cancers by Robotic Approach

A total of 40 patients had undergone surgery for gallbladder cancer using a robotic approach. Upfront radical cholecystectomy was performed in 36 patients; 1 patient underwent revision liver wedge resection with periportal lymphadenectomy, and 3 patients had undergone simple cholecystectomy for polyp in the gallbladder, with intraoperative frozen section being negative for malignancy, but the final histopathology report was suggestive of pT1a disease, hence they were not offered completion surgery.

Pathological outcomes are shown in Table 4.

**Table 4:** Pathological outcomes in GB

T stage	
T1	10
T2	24
T3	6
N stage	
N*x*	3
N0	29
N1	7
N2	1
Median lymph node harvest	8 (2–22)
Margins positivity	1

The median lymph nodal yield was 7 (2–22) in patients who underwent lymph nodal dissection for gallbladder cancer. Only one patient who had undergone radical cholecystectomy had margin positivity over the liver parenchymal side focally.

### Survival Analysis

**Fig. 5:**
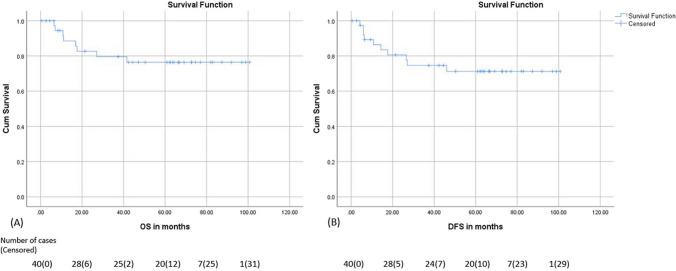
Kaplan–Meir curves depicting (**A**) Overall Survival (OS) and (**B**) Disease Free Survival (DFS)

The median follow-up was 63.9 (0.49–100.67) (IQR = 37.76) months. One-, 3-, and 5-year OS were 88.5, 79.6, and 76.4%, respectively, and 1-, 3-, and 5-year DFS were 86.4, 74.6, and 71.3%, respectively (Fig. 5). At the time of the last follow-up, 7 patients had recurrence (3 peritoneal metastases, 2 hepatic metastases, and 2 with para-aortic nodal recurrence), and 30 patients were alive and disease-free.

## Discussion

In the field of surgical oncology, the open approach has traditionally been considered the gold standard. However, over the past two decades, minimally invasive surgery has proven its advantages over open surgery in terms of short-term outcomes like lesser postoperative pain and shorter hospital stays. Hepatobiliary surgery has its challenges and is associated with significant morbidity and mortality. In the past decade, many retrospective studies have supported the laparoscopic approach as being advantageous to open surgery in terms of lower blood loss, shorter hospital stays, and equivalent oncological outcomes [1, 2]. Disadvantages of laparoscopy included an unstable camera, rigid instruments with a reduced degree of freedom, hand tremors, compromised ergonomics, and difficulty suturing in hard-to-reach locations [5]. The robotic system was designed to circumvent the limitations of laparoscopic surgery. The robotic platform offers certain advantages over the laparoscopic approach in terms of superior dexterity, a stable operating platform, a surgeon-controlled camera, and magnified three-dimensional vision. The Da Vinci Xi system by Intuitive Surgical provides a platform that allows the movement of arms relative to its base and aids in multi-quadrant or multi-cavity surgery without the need for re-docking the arms. Moreover, it has been shown that robotic platforms have superior ergonomics that reduce physical workload and are less strenuous for surgeons [6, 7].

Wakabayashi et al. from Japan reported their earlier experience of robotic surgery in 52 patients, of whom 28 were for hepatobiliary and pancreatic surgery, including both benign and malignant [8]. Giulianotti et al. from Italy reported their initial results of 70 robotic liver resections [9], where a majority of patients (60%) had malignancy and 38.5% underwent major hepatectomy with a conversion rate of 5.7%. Recently, Sucandy et al. reported a large series of 100 robotic hepatectomies. The majority of patients in all these series underwent minor hepatectomies, like in the present series, wherein 32/36 (88.8%) patients underwent left lateral hepatectomy and non-anatomical resection [10].

In the present series, all patients with liver resections underwent R0 resection. For non-anatomical resections, indocyanine green was given at a dose of 0.2 mg/kg to patients at least 5 days before surgery to facilitate intraoperative identification of tumors in firefly mode on the robotic console. We have utilized this technique in 2 patients, and we believe it helps in assessing the margins intraoperatively during parenchymal transection, thereby facilitating margin-negative resection. To date, there are no satisfactory parenchymal transection devices for robotic resections. Most of the series reports a combination of bipolar and monopolar energy devices along with bedside assistants using harmonic scalpel, or CUSA [8–11]. Other energy devices commonly used are the vessel sealer and harmonic curved shears [11–13]. A rubber band traction method was described by Yang et al., which involves the simultaneous use of all three robotic arms during parenchymal transection [11]. Therefore, bleeding can be effectively controlled by compression using the third robotic arm or by compression followed by suture ligation. In addition, ICG fluorescence imaging has been used to identify an ischemic demarcation line through a direct or indirect staining technique, which can facilitate transecting an exact anatomical plane during parenchymal transection. However, the bottom line remains meticulous intraparenchymal dissection and tackling intrahepatic vessels as per size, with careful application of clips for larger vessels (> 7 mm).

The current study included 64 (69.6%) patients who underwent surgery for gallbladder pathologies, of which the majority (39 (60.9%)) underwent radial cholecystectomy. Gallbladder carcinoma is typically diagnosed at an advanced stage and has a high possibility of lymphatic metastasis. Adequate lymphadenectomy is essential to improving survival outcomes. Studies have shown that the resection and histologic evaluation of at least six lymph nodes are required to improve the risk stratification of gallbladder cancer [14], and the range of lymphadenectomy should include the posterosuperior pancreatic head lymph nodes (station 13) along the hepatoduodenal ligament (station 12) and the hepatic artery (station 8). The median lymph nodal yield in the present series was 7 (2–22), which adheres to AJCC recommendations of adequate staging. The postoperative outcomes in terms of blood loss, duration of surgery, and hospital stay have been acceptable with no mortality, which is comparable to the available literature [15, 16]. Byun et al. reported their series of 13 patients undergoing robotic radical cholecystectomy from South Korea with lymph node harvest of 7.2 ± 3.1 [15]. Pickens et al. reported a series of 20 patients with similar outcomes with lymph node harvest of 5 (2–15) [16]. They had reported a 2-year survival of 60.5%. Yang et al. reported the outcomes of robotic extended cholecystectomy in 28 patients, with similar short-term outcomes as in the present study [17]. The median follow-up in their study was 16 ± 10 months and 3 years, OS and DFS were 75 and 57.2%, respectively. In the previous publication from the same institute, we reported short-term outcomes with a median follow-up of 9 (1–46) months and a survival of 92.6% and concluded that the robotic approach is safe [18]. In the present series, with a median follow-up of 63 months, the 1-, 3-, and 5-year OS were 88.5, 79.6, and 76.4%, respectively, and the 1-, 3-, and 5-year DFS were 86.4, 74.6, and 71.3%, respectively. There was initial resistance while offering a minimal access approach in radical cholecystectomy given the difficulty in achieving adequate lymphadenectomy and the chances of gallbladder perforation leading to bile spillage and direct upstaging, increasing the chances of peritoneal metastasis. Case selection is crucial while planning robotic surgery. In the present series, only early diseases as per radiology were selected with no or minimal liver infiltration without radiological periportal lymphadenopathy.

Nine patients underwent conversion to open surgery, primarily due to the need for extrahepatic bile duct excision, dense adhesion in porta hepatis, and iatrogenic injury (Table 1). Consideration for patient safety was the reason for keeping a low threshold for conversion to open surgery. The cost of robotic instrumentation remains the main hindrance to offering this surgery to a large group of patients in our country, despite its technical superiority over traditional laparoscopic surgery.

## Conclusion

Robotic hepatobiliary surgery is feasible and has impressive short-term outcomes. The safety of robotic platforms for carefully selected patients has been demonstrated. The versatility and wide range of motion of robotic instruments are major advantages over laparoscopy. Oncological outcomes in terms of non-inferiority or superiority by direct comparison with the traditional open approach and even the laparoscopic approach are still awaited.

## Data Availability

The datasets generated during and/or analyzed during the current study are available from the corresponding author upon reasonable request.
